# Creation and Assessment of a Bad News Delivery Simulation Curriculum for Pediatric Emergency Medicine Fellows

**DOI:** 10.7759/cureus.595

**Published:** 2016-05-01

**Authors:** Corrie E Chumpitazi, Chris A Rees, Bruno P Chumpitazi, Deborah C Hsu, Cara B Doughty, Martin I Lorin

**Affiliations:** 1 Department of Pediatrics, Baylor College of Medicine; 2 Department of Pediatrics, Section of Gastroenterology, Hepatology, and Nutrition, Baylor College of Medicine; 3 Department of Pediatrics, Section of Emergency Medicine, Baylor College of Medicine

**Keywords:** pediatric emergency medicine, delivering critical news, simulation, standardized patients, education, breaking bad news

## Abstract

Background

Bad news in the context of health care has been broadly defined as significant information that negatively alters people’s perceptions of the present or future. Effectively delivering bad news (DBN) in the setting of the emergency department requires excellent communication skills. Evidence shows that bad news is frequently given inadequately. Studies show that trainees need to devote more time to developing this skill through formalized training. This program’s objectives were to utilize trained standardized patients in a simulation setting to assist pediatric emergency medicine (PEM) fellows in the development of effective, sensitive, and compassionate communication with patients and family members when conveying bad news, and to recognize and respond to the patient/parent’s reaction to such news.

Methods

PEM fellows participated in a novel curriculum utilizing simulated patients (SPs) acting as the patient’s parent and immersive techniques in a realistic and supportive environment. A baseline survey was conducted to ascertain participant demographics and previous experience with simulation and DBN. Experienced, multi-disciplinary faculty participated in a training workshop with the SPs one week prior to course delivery. Three scenarios were developed for bad news delivery. Instructors watched via remote video feed while the fellows individually interacted with the SPs and then participated in a confidential debriefing. Fellows later joined for group debriefing. Fellow characteristics, experience, and self-perceived comfort pre/post-course were collected.

Results

Baseline data demonstrated that 78% of fellows reported DBN two or more times per month. Ninety-three percent of fellows in this study were present during the delivery of news about the death of a child to a parent or family member in the six-month period preceding this course. Fellows’ self-reported comfort level in DBN to a patient/family and dealing with patient and parent emotions improved significantly (p=0.034 and p=0.046, respectively).

Conclusions

Pediatric emergency medicine fellows frequently deliver bad news. A course using SPs was well received by trainees and resulted in improvement in self-assessed skills and comfort. This curriculum provides the opportunity for fellows to receive patient/parent feedback of their communication skills and observations from skilled instructors. This methodology should be considered when creating training curricula for bad news delivery skills.

## Introduction

Bad news in the context of health care has been broadly defined as significant information that negatively alters people’s expectations or perceptions of the present or future [1-2]. Delivering bad news (DBN) in the pediatric emergency department requires enhanced communication skills due to the acute context. While a vital part of medical practice, DBN compassionately is not a part of many training curricula [3], and there is evidence that bad news is frequently delivered inadequately, according to parents of pediatric patients [4]. In Pediatric Emergency Medicine (PEM), physicians are particularly challenged by DBN as they are charged with establishing a compassionate relationship with a family that they likely have not met before the medical crisis and there is little opportunity to prepare for the event [5-6]. A joint statement by the American Academy of Pediatrics and American College of Emergency Physicians stated, “The death of a child in the emergency department is an event with emotional, cultural, procedural, and legal challenges that often distinguish it from other deaths” [7].

When surveyed, patients and their parents report that DBN in an appropriate, clear, and sensitive manner is extremely important to them [8], and that, from the patient’s perspective, physicians often fail to achieve competence in this area [9]. Physicians at all levels of training report that DBN is a cause of stress and that they feel under-prepared for the task [10]. Moreover, physicians admit to feeling a lack of competence when it comes to this type of communication [11], and there is often a limited opportunity for faculty to provide feedback to trainees on communication skills after DBN [12]. However, effective communication skills in critical conversations can be taught [13], and appropriate preparation can make DBN less stressful for the physician [14].

The pediatric literature in DBN has largely focused on Critical Care Medicine, Pediatric Cardiology, and Pediatric Hematology/Oncology with a notable lack in the literature on DBN in PEM [3, 12]. Using standardized patients (SPs) to teach residents and fellows to DBN has been shown to be an effective educational process that provides trainees with interactions that simulate real-life experience [15]. Despite previous interventions aimed to improve communication in critical conversation, there is a significant need for a more universal training [16-17].

The purpose of this study was to assess PEM fellows’ experience and comfort in DBN and to use SPs and immersive techniques to deliver a curriculum designed to train PEM fellows to 1) effectively, sensitively, and compassionately DBN to patients and family members, 2) recognize and respond effectively to patient/family member(s) reactions to bad news, and 3) disclose a medical error or adverse event in an accurate and sensitive manner.

## Materials and methods

This training program was developed through an iterative process by the study investigators. A cross-sectional survey design was employed to collect baseline data to define PEM fellows’ needs in various aspects surrounding DBN. The survey was developed through an iterative process until consensus was achieved. Participant demographics, previous experience with DBN and disclosing medical errors, experience with pronouncing death, and previous training on responding to patient’s emotions were ascertained through the baseline survey. PEM fellows’ personal comfort on various aspects of critical conversations was also ascertained through the questions included in Appendix A.The survey was distributed to all participants via SurveyMonkey®. Written consent was obtained from study participants prior to participation.  

### Participants 

This training program was developed in a PEM fellowship program based at an urban, quaternary care, freestanding children’s hospital with approximately 120,000 annual patient encounters in the ED. A sample of 14/14 PEM fellows in this training program from three different years of fellowship (PGY 4-6) participated in the course. The instruction was delivered in a one-day course, six hours in length.

### Instructors

One week prior to the delivery of this course, seven SPs and six experienced faculty from four different pediatric subspecialties participated in a training workshop on the aims and methodology of the course. SPs and instructors were provided with a detailed case description, learning objectives, time allotment, and expected emotional tone. If there was a planned course of action for the learner and the SP felt the interaction was not following that course, specific instructions were delineated for how to adjust emotional tone, mood, and demeanor. A guide for the follow-up questions the SP should or might ask was written out. A grid was provided with anticipated learner actions and SP responses. This workshop focused on ensuring that faculty and SPs shared expectations of baseline emotional output displayed by SPs in the instructional scenarios and standardized faculty feedback to anticipated responses by fellows to each simulated scenario. Both SPs and instructors were provided with a two-page checklist of actions and suggested language for debriefing based on learner action.    

### Curriculum

The fellows were individually presented with two separate clinical scenarios and one clinical scenario as a group. The first scenario involved delivering the news of a new oncologic diagnosis to the SP acting as the patient’s parent. The second scenario required the disclosure of a medical error. The third scenario involved six PEM fellows in a team-based mock code in which one of the PEM fellows was designated as the code leader and the emergency medical team brought in a mannequin undergoing active cardiopulmonary resuscitation that the team needed to manage, identify futility, and pronounce death. The designated code leader then notified the SP who was present during the resuscitation efforts of the child’s death. Instructors watched via remote live video feed while the fellows interacted with the SPs.

Immediately following the encounters with the SPs, the fellows participated in a confidential debriefing with the SP and the faculty instructor who watched the scenario remotely via video feed. Faculty members conducted debriefings with both the SP and the PEM fellow using the “debriefing with good judgment” framework [18]. The participating faculty members were trained on the “debriefing with good judgment” methodology prior through our hospital simulation instructor course and concepts were reiterated during the training session. The feedback provided in these sessions was meant to be strictly educational and non-evaluative. A debriefing script was provided for each case. For example, the first checklist item was “the learner introduced himself by name.” The options for the SP response included: “I felt that you introduced yourself clearly, seemed calm, confident, and appropriately serious;” or “I felt that you were not clear in your role, and you seemed uncomfortable or too cheerful;” or “I felt that you did not introduce yourself.”

Immediately following the confidential debrief, all six fellows in each individual session participated in a group debriefing also to reflect more broadly on their actions, thought processes, and emotional states. During the debriefing sessions, PEM fellows analyzed their actions, thought processes, and emotional states, allowing for self-reflection to improve future performance. The group debriefing similarly used the “debriefing with good judgment” approach and allowed for a reflection phase, in which PEM fellows were asked how the SP responded and how they responded. Group learners were asked to rate their maximum level of anxiety or discomfort during the encounter before delving in, to understand what caused the discomfort and their feelings as they progressed through the encounter. The learners were also asked how accurate they were in perceiving the SPs reaction. In addition to the simulated scenarios, there were two, hour-long lectures on medical error disclosure and how to DBN (Table 1).

**Table 1:** Learning Objectives for Cases and Lectures in the Delivering Bad News for Pediatric Emergency Medicine Fellows Curriculum.

Theme	Learning Objectives	Lecture Description	Description of Case(s)
Medical Error Disclosure	The learner should be able to:	Didactic session covering the approaches to disclosing medical errors	12-month-old male with Wolf-Parkinson-White syndrome and intermittent episodes of supraventricular tachycardia. He was seen in the Emergency Department (ED) last week and his medication doses were increased by the learner. Today he was brought back to the emergency department with weakness and electrocardiogram findings of medication toxicity. Shortly after arrival, he seizes and is admitted to the cardiovascular intensive care unit. The learner must disclose the medication error to the parent(s).
	1. Define and discuss what constitutes a disclosable medical error or adverse event.		
	2. List and discuss the principles of team decision making in regards to what, how, and by whom disclosure should be done.		
	3. Appreciate the importance of disclosure and apology, when appropriate, and the pitfalls of blame.		
	4. Disclose the error, or event, sensitively and accurately.		
	5. Express and convey compassion.		
	6. Convey a sincere apology to the patient.		
	7. Recognize and assess the patient’s reaction to the error or event and to its consequences.		
	8. Respond effectively to the patient’s reaction and emotional state.		
	9. Recognize and manage his or her own reaction to the medical error and to the patient’s reaction.		
	10. Recognize the need for ongoing dialogue and support after conveying bad news.		
Nuts and Bolts of Delivering Bad News	The learner should be able to:	Didactic lecture covering the approaches to delivering bad news	*New Diagnosis:* 2-year-old girl well until 1 month prior, presents to the ED with increasing headaches and morning vomiting. Head imaging reveals an aggressive brain tumor. The learner must share the news with parent(s) and discuss the plan.
	1. List and discuss the essential steps in delivering bad news.		
	2. List and discuss the principles of team function in regards to delivering bad news and conveying the idea of team to the patient.		
	3. Convey bad news to the patient in an accurate, supportive, sensitive and compassionate manner.		*High Fidelity Simulation:* 15-year-old male biking home from school and hit by a truck sustaining massive head injury. He is transported to the emergency department with ongoing cardiopulmonary resuscitation. The learner must mobilize the team and continue the resuscitation on arrival (SimMan® 3G, Laerdal). The learner must update the mother when she arrives to the ED, discuss cessation of resuscitation, and conduct the death pronouncement.
	4. Recognize and assess the patient’s reaction to bad news.		
	5. Respond effectively to the upset or distraught patient.		
	6. Recognize and manage his or her own reaction to the bad news and to the patient’s reaction.		
Question and Answer Session on Medical Error Disclosure with Legal Counsel	The learner should be able to:	--	--
	1. Ask questions to legal counsel about the implications of delivering bad news, local resources, and related issues.		

### Workshop Evaluation and Analysis

To assess the sustainability of PEM fellows’ change in comfort in DBN, three months after participation in the course, fellows completed a post-course satisfaction and comfort survey containing the same series of questions as found in Appendix A. The questions used in the post-course satisfaction survey were similar to the questions distributed through SurveyMonkey®. The measure of primary outcome was fellows’ self-perceived change in comfort in DBN. Face and criterion validity were assessed by piloting the survey with former fellows, that were not taking the actual course. Descriptive statistics were calculated for baseline data. Student t-test was performed to compare mean Likert scores on pre- and post-test comfort surveys as parametric statistics at times can be used with Likert data, with small sample sizes, and with non-normal data [19]. A p-value of <0.05 was considered statistically significant. All data analysis was completed using SPSS version 23 (SPSS, Inc.; Chicago, IL). Effect size was calculated for using the two-sided Wilcoxon signed-rank p values. Our local institutional review board approved the study protocol.

## Results

Five first-year PEM fellows, six second-year fellows, and three third-year fellows, representing 100% of eligible fellows in this fellowship program, participated in the curriculum (Table 2). An average of 6.3 years had passed since the participants graduated from medical school. Seventy-eight percent (11/14) of the fellows had previously received lectures on DBN. Most (64%, 9/14) had participated in role-play scenarios for DBN, and 42% (6/14) had experience with SPs prior to this course. Regarding training on responding to a patient’s emotions prior to this intervention, 71% (10/14) of fellows reported having had formal lectures, 57% (8/14) participated in role-play scenarios and also had directly observed clinicians DBN, and 50% (7/14) had experience with SPs.

**Table 2:** Trainee Demographics for Delivering Bad News Curriculum for Pediatric Emergency Medicine Fellows.

Post-Graduate Year	Number (%)
PGY- 4	5 (36)
PGY- 5	6 (43)
PGY- 6	3 (21)
Sex (female)	9 (64)
Race	
White, non-Hispanic	5 (36)
Hispanic	2 (14)
Asian/Pacific Islander	6 (43)
Other	1 (7)

Fellows reported DBN to a patient or a family a mean of five times per month (range 1-20). Ninety-three percent (13/14) of PEM fellows in this study were present during the delivery of news about the death of a child to a parent or family member in the six month period preceding this study, totaling 29 separate events. Fourteen (48%) of these events were with non-English speaking families and 57% (8/14) of the surveyed PEM fellows were present during such events. PEM fellows reported use of interpreters for 100% of those cases. Seventy-eight percent (11/14) of fellows reported DBN two or more times per month. Sixty-four percent (9/14) had personally led a resuscitation that resulted in a death pronouncement. Seventy-one percent (10/14) of fellows had been present during the delivery of news regarding a medical error.

One hundred percent (14/14) of the PEM fellows completed the pre-test and 85.7% (12/14) responded to the post-test. Pre- and post-test mean Likert scores regarding fellows’ level of comfort in specific components of DBN are shown in Table 3. There was no statistically significant change in fellows’ self-perceived ability to discuss resuscitation status, notify a family of the death of a child, to disclose a medical error, or to have a consistent approach to DBN. There was, however, a significant improvement in fellows’ self-reported comfort level in DBN to a patient or family member, and in dealing with patient and parent emotions.

**Table 3:** Pediatric Emergency Medicine Fellows’ reported comfort levels related to delivering bad news pre- and post-course responses. *SD=standard deviation Scale: 5 point Likert scale where 1 = Very Uncomfortable, 2 = Uncomfortable, 3= Neither Comfortable nor Uncomfortable, 4= Comfortable, 5 = Very Comfortable. Ϯ Effect size calculated with two-sided Wilcoxon signed-rank p values

Fellows’ Reported Level of Comfort to:	Pre-Course Mean (*SD)	Post-Course Mean (*SD)	p-value	Effect Size ϯ
Deliver bad news to a patient of family	3.58 (0.9)	4.25 (0.452)	0.034	0.43
Deal with patient/family emotions	3.5 (0.674)	3.83 (0.577)	0.046	0.41
Have a consistent approach to the delivery of bad news	3.5 (1.087)	4.08 (0.515)	0.059	0.33
Discuss resuscitation status	3.25 (0.866)	3.58 (0.793)	0.102	0.39
Notify a family of the death of a child in the emergency department	3.08 (1.084)	3.5 (1.087)	0.102	0.33
Disclose a medical error	2.92 (0.793)	3.0 (1.044)	0.742	0.07

## Discussion

Identifying an effective educational curriculum to help PEM trainees learn how to DBN is vitally important to training competent and compassionate physicians. This study demonstrates that an intervention utilizing SPs and debriefing can improve PEM fellows’ comfort in DBN to patients and families and in dealing with patient and parent emotions. This curriculum provides fellows with timely feedback about their communication skills from SPs and skilled physician instructors, allowing fellows to improve their patient-centered communication, which has been associated with patient perceptions of less dominant and more appropriate physicians [20]. It is important for PEM fellows to have formal training in DBN as they transition to attending physicians as, in one study, the majority of surveyed parents felt that it was the attending physician's responsibility to inform the family that the child had died [21].

Similar to previous studies, this study demonstrates that SPs can be used to educate physicians in training [22] and our study resulted in improved self-perceived efficacy among PEM fellows. Few programs have used structured skill assessments in DBN [23]. This intervention improved fellows’ comfort in, and perceived ability to DBN, though a statistically significant improvement was not observed in participants’ self-perceived ability to discuss resuscitation status, notify a family of the death of a child, or to disclose a medical error, which may require more time and clinical experience to attain improved self-perceived ability beyond a one-day training session. In comparison to other training programs that last several days [24-25], this intervention demonstrated improved comfort with DBN among trainees after just one day of training.

Though nationwide the incidence of death in the emergency department among children less than 18 years of age is 1/15,000 [26], this study demonstrated that 94% of PEM fellows at our large, quaternary care institution were present for the death of a child in the emergency department during the six-month period preceding this study. Other studies have shown that clinical experience has a limited effect in improving a physician’s ability in DBN [27], highlighting the need for further education throughout graduate and undergraduate medical education.

While unexpected and untimely patient death is challenging for the physician and healthcare team, and oftentimes emotionally and psychologically wrenching [28], this intervention demonstrated an improvement in fellows’ comfort in DBN sustained over the course of three months. However, the long-term sustainability of this program and others aimed at improving skills in DBN should be further studied [29]. Moreover, future studies should be done to objectively measure PEM fellows’ communication skills, using such instruments as the Gap-Kalamazoo Assessment tool [30], which would result in higher Kirkpatrick levels of data.

This study has some limitations. The SPs were highly trained, paid actors, thus this course may be cost-prohibitive for some programs. The primary outcome measure was fellows’ self-perceived change; however, due to the nature of DBN, it is difficult to completely eliminate subjectivity in evaluating such a training program. Future studies might use validated checklists to assess performance in the simulated setting. All fellows received this educational intervention, as we felt it was unethical to withhold this education, thus there was not a control group. Though we included all PEM fellows at our institution, our sample size was only 14 which limited our ability to elucidate significant differences on sub-group analyses. While nearly half of the pronouncements of the death of a child in the ED were done with non-English speaking families, we did not include cases or didactic sessions on DBN with such families. Further studies are needed to develop such curricula for DBN with non-English speaking families. Lastly, this was a single center study, which may limit the generalizability to different graduate medical education and clinical environments.

## Conclusions

PEM fellows at this training program frequently deliver bad news, including informing parents of the death of their child. This curriculum resulted in increased comfort in delivering bad news among PEM fellows. This multi-model curriculum offers confidential, individual feedback from SPs and trained instructors, as well as small team-based simulation and group discussion. This curriculum offers a format that is adaptable to numerous critical conversations and can be applied to a variety of disciplines.

## Appendices

Appendix A

1. What do you find the most difficult task in breaking bad news?
  1. Discussing new diagnosis
  2. Telling patient/parent about recurrence/progression
  3. Discussing resuscitation status (e.g. do not resuscitate)
  4. Involving family/patient in the decision-making process
  5. Discussion with siblings regarding the news
  6. Other _____________________________________
2. How prepared do you feel to break bad news to a patient or their family members?

         1   Very unprepared

         2  Unprepared

         3  Neither prepared or unprepared

         4  Prepared

         5  Very prepared

3. How would you rate your own comfort in dealing with patient/parents emotions (e.g. crying, anger, denial, etc.)?

         1   Very uncomfortable

         2  Uncomfortable

         3  Neither comfortable or uncomfortable

         4  Comfortable

         5  Very comfortable

4. How would you rate your own comfort in discussing code/do not resuscitate (DNR) status in the Emergency Center with a patient or family member?

         1   Very uncomfortable

         2  Uncomfortable

         3  Neither comfortable or uncomfortable

         4  Comfortable

         5  Very comfortable

5. Do I have a consistent approach to the delivery of bad news in my patients/families?

         1   Very inconsistent

         2  Inconsistent

         3  Neither consistent or inconsistent

         4  Consistent

         5  Very consistent

6. How would you rate your own comfort level in notifying a family of the death of a child in the Emergency Center?

         1   Very uncomfortable

         2  Uncomfortable

         3  Neither comfortable or uncomfortable

         4  Comfortable

         5  Very comfortable

7. How would you rate your own comfort level in discussing/disclosing a medical error in the Emergency Center?

         1   Very uncomfortable

         2  Uncomfortable

         3  Neither comfortable or uncomfortable

         4  Comfortable

         5  Very comfortable
